# Respondent-driven sampling on the Thailand-Cambodia border. I. Can malaria cases be contained in mobile migrant workers?

**DOI:** 10.1186/1475-2875-10-120

**Published:** 2011-05-10

**Authors:** Amnat Khamsiriwatchara, Piyaporn Wangroongsarb, Julie Thwing, James Eliades, Wichai Satimai, Charles Delacollette, Jaranit Kaewkungwal

**Affiliations:** 1 Center of Excellence for Biomedical and Public Health Informatics (BIOPHICS), Bangkok, Thailand; 2 Bureau for Vector-borne Diseases, Ministry of Public Health, Bangkok, Thailand; 3 Centers for Disease Control and Prevention, CDC, Atlanta, USA; 4 World Health Organization, Mekong Malaria Programme, c/o Faculty of Tropical Medicine, Mahidol University; 420/6, Rajvithi Rd, Bangkok 10400, Thailand

## Abstract

**Background:**

Reliable information on mobility patterns of migrants is a crucial part of the strategy to contain the spread of artemisinin-resistant malaria parasites in South-East Asia, and may also be helpful to efforts to address other public health problems for migrants and members of host communities. In order to limit the spread of malarial drug resistance, the malaria prevention and control programme will need to devise strategies to reach cross-border and mobile migrant populations.

**Methodology:**

The Respondent-driven sampling (RDS) method was used to survey migrant workers from Cambodia and Myanmar, both registered and undocumented, in three Thai provinces on the Thailand-Cambodia border in close proximity to areas with documented artemisinin-resistant malaria parasites. 1,719 participants (828 Cambodian and 891 Myanmar migrants) were recruited. Subpopulations of migrant workers were analysed using the Thailand Ministry of Health classification based on length of residence in Thailand of greater than six months (long-term, or M1) or less than six months (short-term, or M2). Key information collected on the structured questionnaire included patterns of mobility and migration, demographic characteristics, treatment-seeking behaviours, and knowledge, perceptions, and practices about malaria.

**Results:**

Workers from Cambodia came from provinces across Cambodia, and 22% of Cambodian M1 and 72% of Cambodian M2 migrants had been in Cambodia in the last three months. Less than 6% returned with a frequency of greater than once per month. Of migrants from Cambodia, 32% of M1 and 68% of M2 were planning to return, and named provinces across Cambodia as their likely next destinations. Most workers from Myanmar came from Mon state (86%), had never returned to Myanmar (85%), and only 4% stated plans to return.

**Conclusion:**

Information on migratory patterns of migrants from Myanmar and Cambodia along the malaria endemic Thailand-Cambodian border within the artemisinin resistance containment zone will help target health interventions, including treatment follow-up and surveillance.

## Background

In the past 50 years, the Bureau of Vector Borne Disease (BVBD) of the Thailand Ministry of Public Health has implemented an effective malaria prevention and control programme in Thailand, resulting in large sections of the country becoming malaria-free. However, surveillance data along the endemic border areas with Myanmar and Cambodia still reveal provinces with a high incidence of disease. Population movements along the border areas often bring partially immune or non-immune populations into close proximity to high transmission forested areas, placing them at risk of acquiring malaria. Population movements in these areas, together with the high drug pressure, are considered responsible for the development and spread of drug-resistant *Plasmodium falciparum *[1,2]. In 2008, BVBD in collaboration with the World Health Organization (WHO), developed a programme for the containment of artemisinin-resistant *P. falciparum *malaria along the Cambodia-Thailand border, funded by the Bill and Melinda Gates Foundation (BMGF). It is anticipated that the project will continue until mid-2011 [3], after which extra funding from the Global Fund to Fight AIDS, Tuberculosis, and Malaria Round 9 in Cambodia and Round 10 in Thailand provides continued support to containment activities [2].

A primary objective of the containment project is to strengthen existing cross-sectoral and cross-border efforts to ensure effective prevention and treatment of malaria in migrant and mobile populations. Specific strategies include examining in greater depth their patterns of mobility, working with them to ensure better access to health services, providing tailor-made prevention tools and specific behaviour change and communication strategies, and attempting to incorporate them into routine surveillance systems. Reliable information regarding migrant movements, health status, and care-seeking behaviour is crucial to the development of prevention and control measures along the border areas [4-6].

There is substantial population movement across the Thai-Cambodian border that is largely driven by economics. Migrants from both Cambodia and Myanmar settle for varying periods of time in Thailand, often in search of work. The International Organization of Migration (IOM) reported that Thailand has attracted increasing numbers of migrant workers, mostly from neighbouring countries with over one million registered migrant workers entering the country since 2004 [7]. Channels for migration, in particular labour migration, are defined by the policy of the destination country, usually in response to the demand of domestic labour markets for foreign workers. When the supply through established channels does not match the demand, irregular migration dynamics develop [8], and migrants enter illegally and undocumented. While various government ministries attempt to collect data on migrant workers, they usually have information on the number of registered migrants and those applying for work permits, but little information on the unregistered migrants. The true size of the migrant worker population in Thailand, in particular of irregular migrants, is notoriously difficult to quantify.

Rather than classifying migrant workers as documented or undocumented, the Thailand Ministry of Public Health defines migrants who have been in Thailand for more than six months as M1, and migrants who have been in Thailand for less than six months as M2. Both M1 and M2 migrants are eligible to receive diagnosis and treatment for malaria free of charge at malaria clinics in border zones. Patients who cross the border for a day to seek treatment at the border clinics are counted among the M2. Migrants in Thailand account for a higher proportion of cases than Thai citizens, especially among the M2 migrants [2]. Malaria surveillance in undocumented migrants is challenging, and ensuring treatment compliance and parasite clearance in an environment in which increased parasite clearance times need to be closely monitored is quite difficult in this population. While undocumented and highly mobile workers may receive diagnosis and treatment at malaria clinics along the border areas free of charge, they are often not followed up for compliance and parasite clearance, as is the norm in the provinces targeted by the containment project. Developing innovative strategies for follow-up is critical to the containment effort.

Little is known about migratory patterns along the Thailand-Cambodia border. However, given the hidden and mobile nature of these populations and the difficulty in generating a sampling frame, traditional sampling techniques such as cross sectional community-based surveys or time-location sampling are unable to produce an adequate and statistically valid sample. Respondent driven sampling (RDS) is a modified form of chain-referral or snowball sampling that has been used to sample hidden populations [9-12], and seeks to overcome the biases in traditional snowball sampling. It uses a structured system of incentives to encourage recruitment by peers while limiting the number of individuals each participant can recruit, records the size of each participant's network to weight the sample, and enables calculation of sampling error [9-11], thus allowing for inferences about the characteristics of the population from which the sample is drawn. Pre-survey focus group discussions were held with migrants from Cambodia and Myanmar to assess the feasibility of using RDS. Discussions focused on migrants' links to and relative size of social networks, willingness to participate, and potential barriers to participation such as limitation of local travel due to lack of transportation or poor roads, a burdensome work schedule, or fear of authority figures.

RDS starts with purposeful selection of a few members of the target population, or "seeds", who are selected based on: (1) diversity of demographic and geographic factors; (2) diversity on key outcome variables; and (3) commitment to the goals of the study [12]. Each participant receives an incentive for participating, and a preset number of coupons with which to recruit peers. The recruiter then receives a secondary incentive for each recruited peer who participates. The method continues as seeds recruit first-wave respondents, first-wave respondents recruit second-wave respondents, and continues until the desired sample size is reached. Participants are encouraged to recruit randomly from their personal social networks. Data are collected on the size of the social networks, and the analysis is weighted based on the size of the social networks.

RDS has typically been used to study urban populations, among highly networked communities, such as injection drug users or commercial sex workers. While the migrant population faces similar issues of stigma, and those who are undocumented wish to remain undetected by authorities, questions were raised as to whether the networks in this population would be sufficient to support this methodology. This analysis focuses on the understanding of mobility dynamics of different types of migrant workers in the border area as well as the application of RDS methodology to the migrant population; demographics, malaria prevention and treatment-seeking behaviour of this population are described in another publication [13].

## Methods

### Study area and population

**Figure 1:**
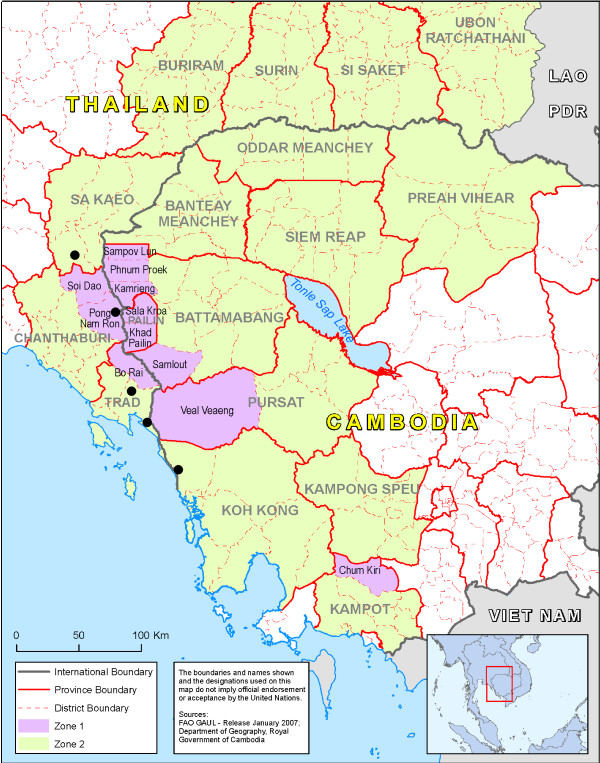
**Location of the 5 study sites**. Falciparum resistance to artemisinin has been documented in the hotspot Zone 1 where intensive containment operations are ongoing.

Five study sites within three of seven provinces along the Thailand-Cambodia border were selected in areas where there were known to be many migrant workers and close by areas where *P. falciparum *resistance to artemisinins has been documented [14]; three sites recruited for Cambodian migrants (one each in Chantaburi, Trat, and Sa Kaeo provinces) and another two sites for Myanmar migrants (both in Trat). The survey was conducted from June to September 2009. Figure 1 shows the five locations in Thailand where surveys were conducted as part of joint Cambodia-Thailand containment operations.

### Sampling

Sample sizes were calculated separately for Myanmar migrants and Cambodian migrants with the assumption that the social networks of the two groups are independent. As the main objective of the study was to estimate the proportions of M1 or M2 among the Cambodian and Myanmar migrant workers in the study areas and there has been no clear sampling frame, the sample sizes were calculated using a conservative target proportion of 50% with a confidence level of 95% and a confidence interval of 0.45, 0.55. A design effect of 2.0 and a non-response rate of 10% were adopted. A sample size of 900 participants for each of the two groups (total 1,800 participants) was required.

### Recruitment

**Figure 2:**
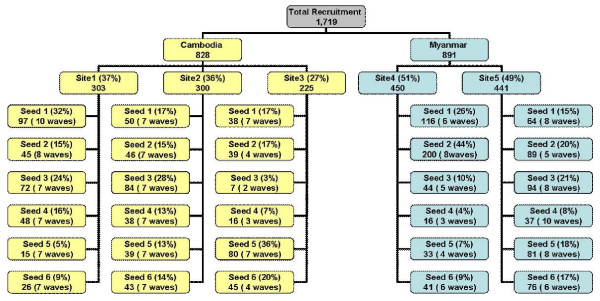
**Recruitment methodology**.

The study staff members were health care workers in the study areas and were trained on RDS methodology. After preliminary interviews with the migrants in the communities, the staff selected six seeds per site for a total of 30 seeds, with a goal of reaching the sample size needed in approximately two months. These seeds were given three uniquely numbered and identifiable coupons to recruit other migrant workers in their networks, who then recruited other migrants in their networks, until the desired sample size was reached. The seeds and their recruits were offered minimal monetary incentives (approximately US$ 10.00 for each recruiter). Each participant was asked to respond to a questionnaire, with questions regarding socio-demographics, migratory pattern, work history, history of malaria infection, health-seeking behaviour, knowledge about malaria and access to health messages. Respondents were asked about the size of their personal networks; both how many people they perceived or knew as migrant workers in their areas, and for weighting, the number of migrant workers from their country of origin who were: (1) aged above 15 and currently residing in the area, (2) personally known and working in this area, (3) personally met in the past 30 days, (4) personally known male workers, (5) personally known female workers, and (6) personally known and recruited into the study. Figure 2 summarizes the recruitment sampling procedures used in Thailand.

### Ethical considerations

The protocol was reviewed by Members of the Communicable Diseases Department of the Ministry of Health and deemed to be exempt from full Institutional Review Board review. Due to the sensitive nature of identity in populations for which RDS methodology is used, consent signed by the participant is not obtained. Following careful explanation of the survey, eligible participants were given the consent form to read or, if necessary, the consent form was read to the survey participant by project staff. All questions were addressed and consenting participants verbally stated that they understood and agreed to all of the items contained in the consent. Following this, a project staff member signed the consent form in the appropriate space.

### Data management

The recruitment was controlled by a coupon management system, which was developed in Microsoft Excel, and this system was used to track the relationships between the recruiters and their recruits. The coupon management system was developed by BIOPHICS and implemented for use at each survey site. After the interview, survey forms were faxed to BIOPHICS via iDatafax data management system. Data quality on the survey forms was checked and reconciled with the coupon management system.

### Statistical analysis

The estimates were performed using the Respondent Driven Sampling Analysis Tool (RDSAT) version 5.6.0 [15]. To explore the information on mobility and working patterns, descriptive statistics and comparisons were done by two types of migrant workers, M1 and M2, among migrants from Cambodia and Myanmar. The social network size was defined as all migrants living in the same community that the participant knew by first name or vice versa, and with whom they had met in the past 30 days. RDSAT was used to calculate weighting of the samples to control for differences in network size and homophily of the population-based estimates for M1 and M2 populations. An analysis of homophily was done to determine the preference of members of a group for connections within one's own group, in this case, M1 and M2. Homophily varies between -1 (completely heterophilous, or having network connections exclusively outside own's group) and +1 (completely homophilous, or having connections exclusively within one's group).

RDSAT software was used to estimate prevalence and confidence intervals for categorical variables other than location. For variables involving locations in Myanmar and Cambodia, un-weighted percentages were reported for each province or state. All analyses were separately performed for Myanmar and Cambodian populations.

## Results

### Recruitment, social networks, and homophily

The initial 30 seeds in this study recruited 1719 study participants during 8 weeks, ranging from 2-10 waves of recruitment and 11-200 participants per seed. The recruitment by seed and site is demonstrated in Figure 2. Among Cambodian migrants, 18 seeds (12 M1 and 6 M2) recruited a total of 828 Cambodian migrants; 350 M1 and 475 M2 (3 not determined). Most were recruited by a neighbor (38.0%) or co-worker (31.1%) among M1 and by a co-worker (77.3%) among M2. Respondents saw their recruiters a median of 20 times in the past month for M1 and 12 times for M2, and had known their recruiters for a median of 10 months for M1 and 2 months for M2. Despite intensive search, only one migrant from Myanmar who had resided in Thailand less than six months was identified to serve as a seed. Among Myanmar migrants, 12 seeds (11 M1 and 1 M2) recruited a total of 891 migrants. In contrast to Cambodian migrants, almost all (871) were M1, and only 19 were M2. Most Myanmar migrants were recruited by friends (M1 - 34.8%, M2 - 21.1%), relatives (M1 - 35.7%, M2 - 42.1%) and neighbors (M1 - 17.0%, M2 - 26.3%). Respondents saw their recruiters a median of 24 times in the past month for M1 and 28 times for M2. Respondents had known their recruiters for a median of 36 months for M1 and 13 months for M2 (Table 1). Cambodian M1 migrants knew of more migrants from their country (78) than either Myanmar M1 (26) or Cambodian M2 (16), a relationship which carried through to individuals known personally (41, 18, and 11, respectively) and those seen in the last 30 days (31, 14, and 11, respectively).

**Table 1:** Unweighted analysis of recruitment patterns and social network sizes

	Cambodian		Myanmar	
Variables	M1 * (n = 350) (%)	M2 ** (n = 475) (%)	M1 * (n = 871) (%)	M2 ** (n = 19) (%)
**Received coupon from**				
Friend	17.1	9.3	34.8	21.1
Spouse	0.6	0.4	2.1	5.3
Relatives	9.7	2.5	35.7	42.1
Neighbour	38.0	4.6	17.0	26.3
Workplace	31.1	77.3	2.5	-
Employer	1.1	1.3	0.8	-
Newly met person	5.7	4.4	7.9	5.3
**Number of times recruiter seen in past month**				
Mean	19.5	16.0	16.9	17.8
Median (IQR)	20 (10-29)	12(10-28)	24(4-28)	28(4-30)
**Frequency of seeing recruiter**				
Daily	51.1	41.7	47.0	57.9
More than weekly	30.6	38.9	10.7	15.8
Weekly	10.3	13.1	25.5	15.8
Monthly	2.0	1.7	7.3	-
Less than monthly	2.6	1.9	9.1	10.5
**Months known recruiter**				
Mean	30.3	4.3	62.4	77.9
Median(IQR)	10(4-28)	2(1-3)	36(12-72)	13(5-180)
**Relationship with recruiter**				
Very close	30.3	11.2	28.4	26.3
Somewhat close	55.4	70.3	49.3	57.9
Not close	11.1	17.3	21.0	15.8
**Reasons for enrollment**				
Compensation	35.4	59.6	20.9	31.6
Friend convincing	40.6	44.2	44.3	47.4
Interested in study	23.1	13.5	26.2	5.3
Have free time	6.6	0.8	10.1	5.3
Other	-	-	3.1	15.8
**Number of labourers from your country aged > 15 in this village (perceived)**				
Mean	78.0	16.2	26.3	11.4
Median(IQR)	40(15-100)	10(8-15)	15(7-30)	10(3-15)
**Of those above, number known personally**				
Mean	41.4	11.0	17.7	10.5
Median(IQR)	21(11-42)	10(7-14)	10(6-20)	8(3-10)
**Of those above, number seen in past 30 days**				
Total				
Mean	30.9	11.4	13.7	8.7
Median(IQR)	20(10-32)	10(7-14)	10(6-20)	7(3-10)
Male				
Mean	16.3	7.0	8.6	5.7
Median(IQR)	10(5-18)	6(4-9)	6(4-10)	5(3-6)
Female				
Mean	16.0	4.7	6.6	4.4
Median(IQR)	10(4-19)	4(2-5)	5(3-9)	4(2-5)
**Number attempted to recruit**				
Mean	4.6	4.6	4.0	3.1
Median(IQR)	3(3-3)	3(3-5)	3(3-3)	3(3-3)

IQR: Interquartile range

* Has lived in Thailand for 6 or more months; ** Has lived in Thailand for less than 6 months

A homophily analysis was conducted to determine whether M2 networks from Myanmar were adequately reached. For migrants from Myanmar, homophily for M1 migrants was 0.213 (some tendency for connections with other M1) and for M2 migrants was -1.0 (connections exclusively with M1). For migrants from Cambodia, homophily was 0.459 for M1 and 0.116 for M2 (both more likely to have in-group connections, M1 more so than M2).

### Migration patterns

Migrants from Myanmar had a longer duration of residence in Thailand than migrants from Cambodia, with a median duration of residence of 87 months in M1 migrants from Myanmar compared to 62 months in M1 migrants from Cambodia. Among M2 migrants, those from Myanmar had been in Thailand 4.5 months, compared to 2.6 months for those from Cambodia.

Long term migrants were more likely to have crossed the border by themselves (among Cambodians, 49% of M1 vs. 35% of M2, and among those from Myanmar 45% of M1 vs. 34% of M2). Long term Cambodian migrants had been helped by relatives (19%), friends (16%), and employers (27%), while 57% of short term Cambodian migrants were helped by employers. Among migrants from Myanmar, 32% of M1 and 41% of M2 had used the services of a broker, compared to 1% of Cambodians. Migrants from Myanmar were also helped by relatives (19% of M1 and 26% of M2). Cambodian M1 migrants were least likely to have paid money upfront to come to Thailand (36%), compared to 53% of Cambodian M2, 58% of Myanmar M1, and 76% of Myanmar M2 (Table 2).

**Table 2:** Mobility patterns of migrant workers from Cambodia and Myanmar, RDS-weighted

Variables	Cambodian		Myanmar	
	M1 * (n = 350) (%)	M2 ** (n = 475) (%)	M1 * (n = 871) (%)	M2 ** (n = 19) (%)
**Duration of stay in Thailand (months)**				
Mean	61.5	2.6	86.8	4.5
Median(IQR)	23(12-60)	2(1-3)	72(33-113)	4 (3-5)
**Border crossing assistance**				
None - by self	49.0% (42.1-56.8)	34.8% (30.9-39.7)	45.2% (41.0-48.7)	33.6% (9.6-58.6)
Broker	1.0% (0.1-2.2)	1.1% (0.2-2.0)	32.2% (28.3-36.3)	41.4% (10.7-67.6)
Relative	18.6% (13.1-22.3)	10% (7.3-12.8)	18.8% (16.1-21.4)	25.9% (8.5-50.7)
Friend	15.5% (10.9-20.2)	10.7% (8.3-13.3)	2.9% (1.7-4.3)	---
Employer	26.8% (18.9-35.9)	56.5% (50.2-61.8)	0.1% (0.0-0.3)	---
Other	2.4% (1.1-3.3)	0.1% (0.0-0.2)	9.6% (6.6-12.7)	---
**Paid money upfront to enter**	36.3% (30.6-46.5)	52.9% (46.0-58.3)	58.2% (54.1-62.2)	76.3% (50.7-100)
**Province of residence before here same as birthplace**	87.5% (82.9-92.0)	90.8% (87.5-94.1)	70.6% (66.6-74.6)	85.7% (67.9-100)
**Return to home country**				
≥ once per week	0.5% (0.0-0.3)	1.3% (0.3-1.9)	---	---
≥ once per month	4.1% (1.2-6.2)	4.2% (2.5-6.0)	---	---
Every 2-3 months	20.6% (12.9-24.7)	32.0% (26.5-36.0)	---	---
Every 6 months	15.6% (10.9-20.4)	26.1% (20.2-29.6)	0.1%(0.0-0.6)	---
Once per year	23.1% (17.6-32.9)	---	2.1%(1.2-3.3)	---
< once per year	5.7% (3.3-9.3)	---	5.3% (3.8-7.2)	---
< once per 5 years	1.6% (0.3-2.6)	---	7.3% (5.5-9.1)	---
Have not been back	28.7% (23.9-37.1)	35.5% (32.0-44.2)	85.2% (82.1-87.8)	100%
**Reasons for returning**				
Visit friends/family	35.5% (29.4-41.8)	23.9% (19.6-27.2)	22.5% (19.3-25.7)	---
Traditional / national holiday	26.6% (22.2-41.0)	51.5% (13.8-56.1)	1.3% (0.402.4)	---
Family event	3.4% (1.4-5.4)	2.2% (0.6-4.2)	0.2% (0.0-0.5)	---
Work	3.7% (1.3-6.7)	1.9% (0.3-3.9)	---	---
Buying/selling (market)	---	---	---	---
Other	5.7% (0.3-8.5)	1.2% (0.2-2.2)	5.3% (3.7-7.2)	---
**Transportation mechanism**				
Bus	8.6% (6.0-11.6)	0.6% (0.1-1.5)	12.3% (10.5-15.2)	---
Taxi	4.8% (1.0-9.0)	0.3% (0.0-0.8)	---	---
Personal vehicle	30.1% (20.3-43.4)	58.7% (48.5-66.6)	0.1% (0.0-0.3)	---
Hired private vehicle	30.1% (23.6-35.4)	6.3% (4.0-8.7)	4.9% (3.3-6.4)	---
Other	0.9% (0.1-1.9)	3.% (1.6-5.6)	13.4% (10.7-16.0)	---
**Frequency of migration**				
In Cambodia in past 3 months	21.9% (15.7-28.2)	72.4% (67.0-77.7)	---	---
Not returned in last 3 months	78.1% (71.8-84.3)	27.6% (22.3-33..0)	---	---
**Plans to return**				
< 6 months	3.9% (0.6-7.1)	25.9% (20.9-30.9)	4.3% (3.1-6.4)	1.4% (0.0-5.4)
≥ 6 months	16.9% (10.5-23.4)	28.4% (23.5-33.3)	---	---
No plans to return	79.2% (72.4-86.1)	45.7% (40.3-51.2)	81.7% (78.5-84.9)	88.3% (75.4-100)
**Median expense of returning** **(32 B = 1 USD)**	(212-501)	(58-140)	(3408-4440)	---
**Plans for next move**				
Myanmar	---	---	4.3% (3.1-6.4)	1.4% (0.0-5.4)
Cambodia	31.6% (23.4-42.6)	68.1% (60.5-74.2)	---	---
No plan to move	36.0% (28.0-41.7)	22.1% (17.5-28.7)	81.7% (78.5-84.9)	88.3% (75.4-100)
Don't know	32.4% (25.8-40.1)	9.3% (6.6-11.9)	13.8% (10.5-16.5)	10.3% (0.0-22.7)
**Reason to move**				
Want a different job	3.2% (1.1-5.9)	9.7% (7.2-12.3)	0.2% (0.0-0.5)	---
My work is finished	11.4% (7.1-14.9)	32.7% (27.8-37.4)	0.4% (0.0-0.8)	
My family member's work	3.4% (1.1-6.0)	5.1% (2.9-7.1)	2.3% (1.3-3.2)	4.1% (0.0-10.9)
To live with my family	15.4% (8.5-22.2)	19.1% (14.4-24.8)	1.6% (0.8-2.5)	---
Immigration status expired	----	----	---	---
Other	3.6% (1.3-6.0)	1.8% (0.4-2.6)	1.6% (0.7-2.8)	---

IQR: Interquartile range

* Has lived in Thailand for 6 or more months; ** Has lived in Thailand for less than 6 months

‡ Denominator = total number in that column, thus may be < 100% for questions asked of a subset

Among Cambodian migrants, 5% of M1 and 6% of M2 returned to Cambodia more frequently than once monthly. Among M2, 36% had not returned since their arrival, 32% returned every 2-3 months, and 26% returned every 6 months. Among M1, 21% returned every 2-3 months, 16% returned every six months, 23% returned once annually, and 29% had not returned since their arrival. This pattern was very different for migrants from Myanmar; 85% of M1 and 100% of M2 had never returned to Myanmar. Only 2% reported returning as often as once per year. Of those who returned, the primary reason among long-term migrants from both Cambodia and Myanmar was to visit friends and family. Cambodian M2 migrants were more likely to return for traditional or national holidays. Public transportation was not a popular option for Cambodians returning home; most travelled by personal or hired vehicle (Table 2). While only 22% of M1 return at least every 2-3 months, 72% of M2 have been to Cambodia in the last three months.

In terms of future plans, of Cambodians, 32% of M1 and 68% of M2 planned to return to Cambodia. Only 4% of migrants from Myanmar planned to return. Among M1 migrants, 36% of Cambodians and 82% of those from Myanmar had no plans to move, and an additional 32% of Cambodians and 14% from Myanmar did not know. By comparison, among Cambodian M2, 22% had no plans to move and 9% did not know. The primary reason for an upcoming move was 'work finished' or 'to live with family members (Table 2).

Migration patterns were analysed in terms of province or state of birth, location considered home, location of previous residence, and for Cambodians planning to move on, the location of the next move, aggregating M1 and M2. With the exception of a few north-eastern provinces, Cambodian migrants came from most of the provinces in Cambodia, though the majority came from western Cambodia (Table 3). Most Cambodians came from their province of birth to their current location in Thailand (Table 2), and most still consider Cambodia home. Among migrants from Myanmar, 86% came from the Mon state, on the eastern border of Myanmar, and 24% now consider Thailand home. Migrants from Myanmar had considerably more displacement from their province of birth prior to arriving at their current residence; almost one quarter reported living elsewhere in Thailand before their current residence (Table 4).

**Table 3:** Migration patterns by province of Cambodian migrants, including both short and long term migrants, unweighted

Province	Province of birth	Province considered home	Prior province of residence	Plans for next move
	N = 808	N = 729	N = 791	N = 310
**In Cambodia**				
BANTEAY MEANCHEY	37 (4.5%)	35 (4.7%)	38 (4.7%)	23 (7.4%)
BATTAMBANG	225 (27.5%)	222 (30.0%)	239 (29.8%)	103 (33.1%)
KAMPONG CHAM	55 (6.7%)	31 (4.2%)	51 (6.4%)	8 (2.6%)
KAMPONG CHHNANG	10 (1.2%)	10 (1.4%)	11 (1.4%)	3 (1.0%)
KAMPONG SOM	1 (0.1%)	1 (0.1%)	2 (0.2%)	0.0%
KAMPONG SPEU	43 (5.3%)	35 (4.7%)	37 (4.6%)	30 (9.6%)
KAMPONG THOM	36 (4.4%)	33 (4.5%)	32 (4.0%)	27 (8.7%)
KAMPOT	102 (12.5%)	58 (7.8%)	86 (10.7%)	14 (4.5%)
KANDAL	9 (1.1%)	4 (0.5%)	7 (0.9%)	2 (0.6%)
KOH KONG	60 (7.3%)	10 (1.4%)	88 (11.0%)	2 (0.6%)
KRATIE	7 (0.9%)	7 (0.9%)	7 (0.9%)	1 (0.3%)
ODDAR MEANCHEY	6 (0.7%)	5 (0.7%)	6 (0.7%)	5 (1.6%)
PHNOM PENH	22 (2.7%)	13 (1.8%)	17 (2.1%)	8 (2.6%)
PREY VENG	22 (2.7%)	12 (1.6%)	14 (1.7%)	2 (0.6%)
PURSAT	25 (3.1%)	24 (3.2%)	23 (2.9%)	12 (3.9%)
SIEM REAP	77 (9.4%)	74 (10.0%)	70 (8.7%)	57 (18.3%)
SIHANOUKVILLE	0.0%	2 (0.3%)	1 (0.1%)	0.0%
SVAY RIENG	10 (1.2%)	8 (1.1%)	7 (0.9%)	4 (1.3%)
TAKEO	60 (7.3%)	46 (6.2%)	53 (6.6%)	6 (1.9%)
**In Thailand**				
CHANTABURI	0.0%	14 (1.9%)	0.0%	1 (0.3%)
TRAT	0.0%	84 (11.4%)	0.0%	0.0%

**Table 4:** Migration patterns by province of migrants from Myanmar, including both short and long term migrants, unweighted

Province or state	Province of birth	Province considered home	Prior province of residence
	N = 870	N = 852	N = 879
**In Myanmar**			
MON	747 (85.9%)	546 (64.1%)	543 (61.8%)
BAGO	12 (1.4%)	9 (1.1%)	8 (0.9%)
TANINTHARYI	32 (3.7%)	24 (2.8%)	26 (3.0%)
RANGOON	25 (2.9%)	22 (2.6%)	10 (1.1%)
OTHER	54 (6.2%)	46 (5.4%)	83 (9.4%)
**In Thailand**			
BANGKOK	0.0%	0.0%	16 (1.8%)
KANCHANABURI	0.0%	0.0%	18 (2.0%)
RANONG	0.0%	0.0%	7 (0.8%)
SAMUT SAKHON	0.0%	0.0%	32 (3.6%)
TAK	0.0%	0.0%	10 (1.1%)
TRAT	0.0%	205 (24.1%)	77 (8.8%)
OTHER	0.0%	0.0%	39 (5.5%)

## Discussion

Significant differences in the migration patterns of migrant workers were found from Cambodia and Myanmar on the Thai-Cambodia border. Migrants from Myanmar had a longer duration of residence in Thailand, rarely if ever returned to Myanmar, and were more likely to consider Thailand home than Cambodians. The vast majority had came from Mon state, had used the services of a broker to enter Thailand, and were more likely to have lived in other locations in Thailand before arriving in their current location. The majority of migrants from Cambodia are short-term migrants, and more than half have been in Cambodia in the last three months, usually for holidays or to visit friends and family. While less than a third of Cambodians who have lived in Thailand for more than six months had plans to return to Cambodia, more than two-thirds of short-term migrants had plans to return, and almost three quarters had been in Cambodia in the past three months. The provinces listed as province of origin and of next destination covered most of Cambodia, though the majority were western and central provinces.

RDS has historically been used to study other hidden or difficult-to-reach populations, including men who had sex with men, commercial sex workers, and injection drug users, often in the context of HIV prevention, and has been shown to be a flexible and robust method that can produce a sample representative of the heterogeneity of hidden populations [16-19]. This methodology was successful in recruiting a sufficient sample of both Cambodian and Myanmar migrants living and working on the Thai-Cambodia border, despite initial concerns that social networks might not be large enough, and that restrictions in travel would hinder the ability to recruit new participants. While the overall design of this study was very similar to RDS studies done with other populations, several additional factors in recruiting this population had to be taken into account. First, while most RDS studies have usually been done in urban areas, this population is largely rural, and issues around funding for transportation had to be considered. Location of the study sites was extremely important to assure access. Due to language barriers in these migrant populations, translators were necessary. Because the agricultural work that many migrants come to do is highly seasonal, it was critical to time the study with the peak in the need for agricultural work.

In the Cambodian community, there were strong networks between recent and more long-term migrants, resulting in the recruitment of more short-term (M2) migrants than long-term (M1) despite starting with more M1 seeds. In the Myanmar community, very few short-term migrants were recruited, which was confirmed by discussions with key informants stating that there were in fact few short-term migrants from Myanmar, perhaps reflecting the amount of time required for migrants to travel from the western to the eastern side of Thailand. In addition, the homophily analysis showed that M2 migrants from Myanmar were recruited exclusively by and exclusively recruited M1 migrants, showing that they are well-integrated into the long-term migrant community and confirming that they are likely few in number. While RDS methodology is not immune from sampling bias [20,21], and discrepancies between network compositions or the recruitment behaviours (including social cohesion in recruiting from only one's own types) may impact the nature of the sample, this does not appear to have been responsible for the preponderance of long-term migrants from Myanmar.

While respondent driven sampling proved to be an effective sampling methodology to study mobile migrant populations, there were some challenges and limitations in the implementation. While not inherent to RDS, staff struggled with the length of the questionnaire, and this as well as the need for interpreters may have limited data quality. All answers were self-reported and not based on observation, possibly resulting in some recall or reporting biases. Blood samples were not collected. They would have given more information not only as to the movement of the human population, but of parasites as well.

The migration patterns demonstrated here have implications for containment of artemisinin resistance. There was a concern that migrants from Myanmar may carry the resistant parasite back to their country of origin, which is highly endemic, has not achieved the levels of malaria control that other SE Asian countries have, and has limited access by international agencies due to the political situation. However, those residing along the Thai-Cambodia border, primarily in Trat, have in fact largely settled in Thailand, and do not return often, if at all. The Cambodian population in the three border provinces of Trat, Chantaburi, and Sa Kaeo differ in three important ways. First, there is considerably more frequent cross-border mobility, with even long-term migrants returning on a regular basis. Second, while migrants come primarily from western and central Cambodia, there has been migration from and back to almost all provinces in Cambodia, some with very low transmission levels and largely non-immune populations. Interestingly, no migrants claimed an origin in Pailin Province, which has typically been thought to be an epicenter of development of anti-malarial resistance. Finally, while most migrants had been in Cambodia in the last three months, very few crossed the border more frequently than monthly. Whether this reflects the true reality of the situation or is due to sampling bias if the networks of those who cross more frequently did not intersect with those of the labourers in this sample, it seems as if the population resides in Thailand long enough to benefit from a treatment follow-up programme. Frequent population movements, both across the Thailand-Cambodia border and from the border area across Cambodia, indicate the need for heightened surveillance for artemisinin tolerance outside what has been designated as the containment zone, as well as close cooperation amongst Thai and Cambodian authorities.

Migration contributes to re-emergence of malaria in previously malaria-free areas [22], and cases of malaria in migrants are often reported among people who recently returned to their countries of origin to visit friends and family [23]. In Thailand, 46% of all malaria cases reported were in short term migrants in 2003; this increased to 55% in 2006 [2]. As national reports do not take into account reports from non-state organizations dealing with migrants, these figures are likely to be an underestimate. As these individuals may then return to homes across Cambodia, they may carry resistant parasites with them. Effective containment depends on identifying and promptly treating all cases of malaria. However, unregistered migrants are in a vulnerable position as they may be subject to arrest and deportation, and do not have health insurance [7], discouraging health care-seeking in Thailand. Furthermore, even if they seek treatment from Thai malaria clinics that do not require insurance, they often do not receive the same follow-up to assure success of treatment, under the assumption that they are too mobile to follow through the 28 days follow-up period. While data were not collected on how frequently migrants changed employers (and potentially residences), these data suggest that only a small minority return to Cambodia at least monthly, and that the majority likely remain long enough to complete a 28-day follow-up period. Case follow-up is crucial for artemisinin resistance containment; further research should be done to elucidate the future movement plans of short-term migrants diagnosed in malaria clinics and determine the best way to assure follow-up.

## Conclusion

Respondent driven sampling methodology was an effective strategy to study the migrant populations from Myanmar and Cambodia on the Thailand-Cambodia border. Findings suggest that while populations from Myanmar are relatively settled, populations from Cambodia return home with relative frequency, not only to the border areas, but to provinces across Cambodia, indicating a need for heightened surveillance of artemisinin resistance across Cambodia. However, migration does not appear so frequent as to render case follow-up impossible, and containment of resistance depends on providing diagnostics, treatment, and follow-up to highly mobile migrants as well as to more settled populations. Population mobility is a fact of global life; addressing the health needs of migrants will not only improve migrant health but also reduce long-term health and social costs and protect public health. This information on the mobility of cross-border migrants on the Thailand-Cambodia border will be valuable in planning effective malaria prevention and control for the border areas and help move forward the programme goals of containment and elimination of the disease.

## Competing interests

The authors declare that they have no competing interests.

## Authors' contributions

All authors were involved in the conception and design of the study and design of the application tools for data collection. PW were in charge of managing the study and monitoring field research activities. CD arranged for technical assistance and consultation of the study conceptual framework. WS was responsible for managing and supervising overall malaria control programme activities. AK monitored field activities under RDS method and extracted data for analysis. AK and JK performed statistical analyses and drafting the manuscript. JT provided support for analysis and drafting of the manuscript, and JE was instrumental in the study design and drafting of the manuscript. All authors read and approved the final manuscript.

## Disclaimer

The findings and conclusions in this report are those of the authors and do not necessarily represent the official position nor of the Centers for Disease Control and Prevention neither of the WHO.
